# Can an Infection Hypothesis Explain the Beta Amyloid Hypothesis of Alzheimer’s Disease?

**DOI:** 10.3389/fnagi.2018.00224

**Published:** 2018-07-24

**Authors:** Tamas Fulop, Jacek M. Witkowski, Karine Bourgade, Abdelouahed Khalil, Echarki Zerif, Anis Larbi, Katsuiku Hirokawa, Graham Pawelec, Christian Bocti, Guy Lacombe, Gilles Dupuis, Eric H. Frost

**Affiliations:** ^1^Division of Geriatrics, Department of Medicine, Research Center on Aging, University of Sherbrooke, Sherbrooke, QC, Canada; ^2^Department of Pathophysiology, Medical University of Gdańsk, Gdańsk, Poland; ^3^Singapore Immunology Network, A^∗^STAR, Biopolis, Singapore, Singapore; ^4^Department of Pathology, Nitobe Memorial Nakano General Hospital, Tokyo, Japan; ^5^Department of Internal Medicine II, Center for Medical Research, University of Tübingen, Tübingen, Germany; ^6^Health Sciences North Research Institute, Greater Sudbury, ON, Canada; ^7^Department of Biochemistry, Graduate Programme of Immunology, University of Sherbrooke, Sherbrooke, QC, Canada; ^8^Department of Microbiology and Infectious Diseases, University of Sherbrooke, Sherbrooke, QC, Canada

**Keywords:** peripheral innate immune system, Alzheimer’s disease, infections, amyloid beta, blood–brain barrier, monocytes/macrophages, biofilms, senile plaques

## Abstract

Alzheimer’s disease (AD) is the most frequent type of dementia. The pathological hallmarks of the disease are extracellular senile plaques composed of beta-amyloid peptide (Aβ) and intracellular neurofibrillary tangles composed of pTau. These findings led to the “beta-amyloid hypothesis” that proposes that Aβ is the major cause of AD. Clinical trials targeting Aβ in the brain have mostly failed, whether they attempted to decrease Aβ production by BACE inhibitors or by antibodies. These failures suggest a need to find new hypotheses to explain AD pathogenesis and generate new targets for intervention to prevent and treat the disease. Many years ago, the “infection hypothesis” was proposed, but received little attention. However, the recent discovery that Aβ is an antimicrobial peptide (AMP) acting against bacteria, fungi, and viruses gives increased credence to an infection hypothesis in the etiology of AD. We and others have shown that microbial infection increases the synthesis of this AMP. Here, we propose that the production of Aβ as an AMP will be beneficial on first microbial challenge but will become progressively detrimental as the infection becomes chronic and reactivates from time to time. Furthermore, we propose that host measures to remove excess Aβ decrease over time due to microglial senescence and microbial biofilm formation. We propose that this biofilm aggregates with Aβ to form the plaques in the brain of AD patients. In this review, we will develop this connection between Infection – Aβ – AD and discuss future possible treatments based on this paradigm.

## Introduction

Alzheimer’s disease (AD) was identified over 100 years ago when Alois Alzheimer and others described the pathological hallmarks of this devastating disease (Alzheimer, 1907; Alzheimer et al., 1995). The histopathological characteristics of AD are extracellular deposition of Aβ and intracellular accumulation of the tau protein in a hyperphosphorylated form (Hanger et al., 2014; Sun et al., 2015) leading to synaptic dysfunction which highly correlates with the cognitive decline (Terry et al., 1991). These findings gave rise to what is called the “amyloid cascade hypothesis of Alzheimer’s disease" (Beyreuther and Masters, 1991; Hardy and Allsop, 1991; Karran and De Strooper, 2016). Neuroinflammation is regularly observed in AD and has been incorporated into the amyloid hypothesis (Rogers et al., 1992; McGeer and McGeer, 2013; Bolós et al., 2017). However, despite the discovery of these pathological hallmarks almost 100 years ago and the belief that they are also at the origin of the disease, no progress has been made in AD treatment (Mehta et al., 2017; Sacks et al., 2017). Thus, a new paradigm is needed to integrate the knowledge accumulated through the decades and which can lead to further discoveries and effective treatments that are so urgently required.

## What Is Alzheimer’s Disease?

### Clinical Aspects

Alzheimer’s disease is one of the most deleterious neurodegenerative diseases known (Tam and Pasternak, 2012). Clinically, AD can be well-defined and distinguished from other dementias. Starting with memory problems, it progresses inexorably toward the total loss of the patient’s identity (Castellani et al., 2010). In our aging society, it has become one of the most disastrous plagues of modern humanity. Whereas the incidence of heart disease and cancer are declining or stable, AD and dementia are expected to almost triple in the next 30 years. However, it is still questionable whether there are as many clinically “pure” forms of AD, presenting only with the typical pathophysiological alterations in the brain cortex and leading to neurodegeneration, as is claimed (Fulop et al., 2013a,b). We still do not know whether most cases diagnosed as AD are in reality a mixed form involving cortical as well as subcortical cognitive changes (Schreiter Gasser et al., 2008), although the recent inclusion of many cardiovascular risk factors seems to support this contention (Gottesman et al., 2017; Falsetti et al., 2018). From the clinical point of view, efforts have been made to redefine AD to accommodate the amyloid hypothesis. Recently, it has been claimed that the initial stages of AD designated as prodromal or pre-clinical stages, or subjective memory complaint (SMC) which still cannot be clinically diagnosed with any certainty, may nevertheless only be detected by changes in the “biomarkers” of AD such as Aβ and pTau levels in the cerebrospinal fluid (McKhann et al., 2011; Reiman et al., 2011; Blennow and Zetterberg, 2018; Ghidoni et al., 2018).

This new and very appealing clinical classification of AD prior to the clinical manifestation of the disease, nevertheless has helped to advance the field, but not exactly as it was intended (Bondi et al., 2017). It has rather stimulated the AD community by making it aware of the fact that AD is not exclusively a disease of the elderly but starts decades before the first clinical symptoms such as mild cognitive impairment (MCI) appear (Le Page et al., 2017). Despite this new classification, all stages of the disease, pre-clinical and clinical, were linked to the prevailing paradigm of the amyloid hypothesis which states that pathological Aβ production induces a neuroinflammatory state which, once crossing a certain threshold, results in overt AD. This new classification has incorporated the notion of neuroinflammation, recognized many years ago, but again linked to Aβ production and deposition, despite the emerging evidence that neuroinflammation actually precedes Aβ deposition as will be discussed later in this review (McManus and Heneka, 2017).

### Pathological Aspects

Alzheimer’s disease was initially described by Alois Alzheimer in a 56-years-old woman who had most probably a familial (hereditary) form of early onset AD according to our present understanding (Alzheimer, 1907; Alzheimer et al., 1995). This form comprises only about 5% of all AD cases, whereas the vast majority are so-called late onset AD, which is more frequent with increasing age (Bekris et al., 2010). Thus, age is considered as one of the most important recognized risk factors for late onset AD (Castellani et al., 2010). From the original observations of Alois Alzheimer on the pathological findings in the cortex of his patient there was a paradigm change in the 80s due to the application of these same pathological findings also as hallmarks of the sporadic form of the disease. Since then, continuing confusion concerning the aetiologies of these two separate pathologies has persisted (Fulop et al., 2013a,b; Bondi et al., 2017). Because the patient described by Alzheimer had amyloid plaques and neurofibrillary tangles, then according to this definition, all AD patients should have the same pathology. Logically this should also be the cause of the disease.

Considerable research efforts have been devoted to the amyloid hypothesis. Initially these efforts were directed toward to identifying what the exact composition of amyloid is and secondly to understand how it is produced. It was established that it originates from amyloid precursor protein (APP) either extracellularly or intracellularly. The normal processing of APP is not amyloidogenic (i.e., does not result in the production of Aβ) and most probably has a physiologic role in synaptic maintenance (Perneczky et al., 2013). However, for some reason(s) which have not been fully investigated, there is a shift in APP processing to an amyloidogenic metabolism and the formation of extracellular Aβ fibrils. These fibrils are the basis of the amyloid plaques already described by Alzheimer. We know that changes in the cellular membranes, and especially the enriched cholesterol contents of their lipid rafts, favors the amyloidogenic pathway (Burns et al., 2006). However, other causes responsible for this amyloidogenic production are known, such as infections but these explanations have not yet gained acceptance in the AD research community (Bourgade et al., 2016a,b).

The most important by-products of amyloidogenic APP metabolism are the beta amyloid peptides 1–42 and 1–40. The most abundant is Aβ1–40, but the most toxic is Aβ1–42 (Galante et al., 2012). These products can be measured in different compartments of the body in addition to the immunopathological determination in the brain. In cerebrospinal fluid and in the blood, it is claimed that Aβ decreases in clinically diagnosed AD. Interestingly, in blood, Aβ was found to be more abundant during the MCI stage of the disease than in clinically diagnosed AD (Camponova et al., 2017). Aβ in blood is also claimed to be a biomarker for the early and very early stages of the disease. Many clinical trials were based on its measurement and the hope that treatment to reduce Aβ levels would consequently prevent the progression to full blown AD (Cummings et al., 2018; Molinuevo et al., 2018). Unfortunately, the many clinical trials that aimed at decreasing Aβ levels in the brain by active or passive vaccination including use of monoclonal antibodies did not result in clinically useful results.

The next issue was to investigate what is the precise mechanism of Aβ production. Huge efforts were devoted to this research as according to the amyloid hypothesis, if we decrease its production even if not completely preventing it, the clinical effects should be notable. The normal mechanism was elucidated and was shown to be driven by α-secretase which produced soluble APP fragments (Chow et al., 2010). The enzymes involved in amyloidogenic Aβ production are β-secretase (BACE) and then γ-secretase acting sequentially (Rivest, 2009; Siegel et al., 2017). Many different compounds that inhibit these secretases were tested in phase II and III clinical trails, but without any clinical success. These failures cast further doubts about continuing to adhere to the amyloid hypothesis.

## Thus, the Question Remains What Could Be the Real Cause of AD?

### The Amyloid Hypothesis

This hypothesis identifying amyloid as the possible cause of AD remains the most popular and widely accepted theory, despite its drawbacks discussed above. This hypothesis states that due to a change in proteostasis, as one of the hallmarks of aging (Witkowski et al., 2017), APP is broken down to create Aβ and when this occurs unopposed, then pathology results. This amyloidogenic proteolysis allows formation of fibrils that deposit extracellularly, kill neurons and form classical senile plaques (McGeer and McGeer, 2013; Bolós et al., 2017). However, it is clear that such senile plaques are commonly present in the brain in the absence of any cognitive pathology. So, how does Aβ act differently in patients compared to healthy people? There are several receptors for Aβ on different cells but mainly on microglia, which are brain macrophages that can engulf Aβ and destroy it. However, with time this process is circumvented, either because Aβ production becomes overwhelmingly increased or because of the “senescence” of the microglia that lose functionality, resulting in Aβ starting to slowly accumulate and deposit in plaques. In parallel, as the microglia are unable to ingest all the Aβ, pathogen or pattern recognition receptors (PRRs) such as the TLRs, CD36, RAGE sense the presence of Aβ and induce a strong inflammatory reaction leading to free radical and pro-inflammatory cytokine production (Su et al., 2016; Venegas and Heneka, 2017; Le Page et al., 2018). This will lead to the well-described neuroinflammation and the destruction of neurons. At the mean-time, the Tau protein which is necessary for the maintenance of axon physiology, becomes hyper-phosphorylated because of this inflammatory process, and forms neurofibrillary tangles, which mauls the structure of neuronal processes leading first to degradation of synapses and consequently to neuron death (Serrano-Pozo et al., 2011; Holtzman et al., 2016; Leyns and Holtzman, 2017). Thus, according to this model, Aβ is at the center stage of AD at all disease stages. However, in the absence of any noticeable success of treatments based on the amyloid hypothesis, competing hypotheses urgently require consideration.

### Vascular Hypothesis

The “vascular hypothesis” has been present since the very early 1990s. It states that the production of Aβ may be a consequence of ischemia occurring in the brain with age. Cerebral amyloid angiopathy (CAA) is a major pathological feature of AD where amyloid spreads and deposits throughout the blood vessel walls in the central nervous system. These pathogenic events induce a specific clinical presentation profile including cerebral hemorrhage, stroke, ischemic infarctions, subarachnoid hemorrhage, seizures, cognitive impairment, and dementia (Bu et al., 2013). Epidemiological studies have shown that several well-established risk factors for AD, including diabetes mellitus, atherosclerosis, stroke, hypertension, transient ischemic attacks, microvessel pathology and smoking, have a vascular component that reduces cerebral perfusion (de la Torre, 2002). In fact, detection of regional cerebral hypoperfusion through neuroimaging techniques can preclinically identify individuals at risk for AD. Further, cerebral hypoperfusion precedes hypometabolism, cognitive decline, and neurodegeneration in AD (de la Torre, 2002). Therefore, disturbance of the cerebrovascular system is likely to be a major contributor to AD pathogenesis. This seems very attractive, but there are many vascular lesions in the brain which do not lead to Aβ production and deposition.

Alzheimer’s disease might originate from either or both of the amyloid and vascular hypotheses together. Indeed, there could be an intertwined form of AD which is a mixed manifestation where the two pathologies co-exist. Initially one may predominate, but at the end they might become indistinguishable. So, while we can recognize that vascular changes may somehow contribute, these are far from explaining all of the pathogenesis of AD.

### Infection Hypothesis

The infection hypothesis was presented as a hypothetical causative explanation even by Alois Alzheimer himself. This hypothesis was discarded but has more recently been “rediscovered” (Itzhaki et al., 2016). What is the evidence that AD may be of infectious origin?

The resurgence of the hypothesis that microorganisms might have an important role in the development of AD was rekindled by the pioneering work of Itzhaki’s group who showed that plaques contain remnants of HSV-1 viral DNA (Wozniak et al., 2007, 2009, 2011; Itzhaki, 2016). This was one of the first attempts to link AD pathological hallmarks to something other than Aβ. The hypothesis proposes that in people infected by HSV-1 (the majority of elderly persons), some show a decline of the immune system with age which enables HSV-1 to migrate from the periphery to the brain, or alternatively, in stressful circumstances, HSV-1 infects the brain directly via the olfactory route. In the latter case, this infection is mild. Once HSV-1 is in the brain, it is able to facilitate several processes which contribute to neuroinflammation (e.g., direct stimulation of TLRs), as well as to direct neuronal cytopathology and ultimately to neural degeneration (e.g., senile plaque formation). There are experimental data supporting the proposal that HSV-1 in the brain directly contributes to the abnormal processing of APP to Aβ and favors their toxic aggregation and also the hyperphosphorylation of Tau (Itzhaki, 2016). Further experimental data suggest that other viruses, such as CMV, may also be involved in the pathogenesis of AD (Lövheim et al., 2018). More recently the presence of antiviral antibodies in epidemiological studies, especially high IgM levels (representing the reactivation of viral infections), has been correlated with the long-term development of AD (Lövheim et al., 2015). Interestingly, these studies imply that the greater the number of different microorganisms detected in the periphery, the greater the probability of developing AD (Lövheim et al., 2018). This suggests that not one single agent, but a community of microorganisms may be involved in triggering AD (Carter, 2017). These experimental data indicate that such agents may act as environmental pathogenic trigger factors interacting with genetic (e.g., APOE4) and immunologic factors (Costa et al., 2017) to explain the heterogeneity of susceptibility to AD.

A second pioneering group of investigators, invoking the infection hypothesis, has suggested a role for spirochetes in the pathogenesis of AD (Miklossy, 2011a,b, 2016; Miklossy and McGeer, 2016). They demonstrated the presence of *Borrelia burgdorferi* in the post-mortem brains of many AD patients and showed that senile plaques are biofilms built by these organisms to protect themselves from host defense mechanisms and to assure their own survival (Miklossy, 2016). These observations provided a new impetus to the infection hypothesis as we will describe below. However, the exact composition and the origin of the amyloids contained in plaques/biofilms has not been precisely determined.

Almost at the same time, Balin et al. (1998, 2008) also made the important observation that *Chlamydophila pneumonia*, an obligate intracellular, Gram negative bacterium was present in post-mortem AD brains. Systemic infection by this pathogen was associated with a fivefold increase in AD occurrence and many AD patients have increased anti-*C. pneumonia* antibody titers in blood. *C. pneumonia* may also enter directly through the olfactory tract, as was described for HSV-1, and infect or colonize different cells of the brain including microglia (Bu et al., 2015). Viable bacteria were detected by one study near the plaques in AD brains (Gérard et al., 2006). Dormant reservoirs of bacteria have increasingly been discovered in the body. It was recently demonstrated albeit not in AD that *Staphylococcus aureus* may survive in some Kupffer cells and as such constitute a dormant reservoir, the reactivation of which may occur at any time when the circumstances become favorable (Surewaard et al., 2016). These infections were related to the ApoE4 genotype, a known genetic risk factor for AD. Since these seminal observations, other bacteria were demonstrated in AD brain such as *Pseudomonas aeruginosa, Escherichia coli, Helicobacter pylori*, and *S. aureus* (Singhrao et al., 2015; Zhan et al., 2016). Of note, great care was taken by these investigators to exclude the possibility that the presence of these microorganisms in the brain was due to post-mortem contamination.

Consistent with these data, it is well-recognized that periodontitis and gingivitis are linked to a higher risk of AD (Singhrao et al., 2015; Pritchard et al., 2017). As these are chronic inflammatory diseases affecting the whole body it is not surprising that they are also associated with cardiovascular disorders, type 2 diabetes mellitus and rheumatoid arthritis (Carter et al., 2017; Kriebel et al., 2018) which, in turn are additional risk factors for the development of AD. What is not known exactly is the pathomechanism of this connection and whether specific pathogens are involved in this association. There are many candidates but none of them have been proven. In this context the role of *Porphyromonas gingivalis* as the “master bacterium” orchestrating the whole community of microorganisms inside the mouth has been strongly evoked (Hajishengallis et al., 2012). This bacterium is able to subvert the role of organ specific inflammatory cells via different virulence factors such as its LPS and gingipains (How et al., 2016; Olsen et al., 2016). The bacteria, as well their molecules (capsular proteins, flagellin, fimbrillin, peptidoglycan, proteases), may be considered as pathogen-associated molecular patterns (PAMPs) and will interact with PRRs such as TLR-2 and TLR-4 resulting in pro-inflammatory cytokine secretion. This in turn could result in a neuroinflammatory state leading to neuronal destruction and the disruption of the blood–brain barrier (BBB) (Zlokovic, 2011; Keaney and Campbell, 2015). It is of note that *P. gingivalis*, as with all the above-mentioned microorganisms, is able to promote Aβ deposition, thus directly linking this infection to AD (Liu et al., 2017; Wu et al., 2017). These experimental data suggest that this is a physiological response of the organism to these microbiological challenges. Furthermore, *P. gingivalis* can disrupt the BBB (van de Haar et al., 2016), which may facilitate the entry of other cells and pathogens into the brain. Once more, the microorganisms of the mouth are also forming biofilms to assure their survival and their virulence (Liu et al., 2017) (immune evasion) and creating a quorum sensing like milieu which may affect the responses induced by each other (Srinivasan et al., 2017). This is of fundamental importance for their survival by protecting them from the host immune system. In summary, probably everybody would have *P. gingivalis* in their brains but depending on its virulence, on their genetic makeup and their susceptibility to develop inflammation, they may or may not be suffering from AD.

Another group of oral bacteria may also play a role in AD, namely Treponema, of which there are several species in the oral microbiome. *Treponema pallidum*, the infectious agent of syphilis, although not an oral treponeme, can invade the brain and provoke a chronic infection leading to neurosyphilis, which has common features with AD, due to its ability to evade the immune system. Oral treponemes have also been found in the brain and may also be able to efficiently evade the immune system and provoke chronic infections.

Recently, mutual influences in the gut–brain axis (bidirectional communication) have been suggested to contribute to the development of AD (Alkasir et al., 2017; Jiang et al., 2017). The microbiota are powerful modulators of whole-body metabolism. Dysbiosis, which can occur with aging, is associated with increased gut permeability that may influence several factors playing a role in the increased incidence of AD with age (Biagi et al., 2010). Most importantly, the dysregulated microbiota (gut, mouth, and nose) may lead to a systemic inflammatory state which impedes the functioning of brain cells including the activation of microglia at the origin of neuroinflammation. A vicious circle is then initiated between the brain and the gut facilitated by the increased BBB disruption (Jiang et al., 2017). Furthermore, this inflammation may also result in the invasion of microbes or microbial products such as LPS and amyloids into the brain (Bhattacharjee and Lukiw, 2013; Hufnagel et al., 2013), contributing to neuroinflammation and the resulting production of Aβ and pTau (Torrent et al., 2012; Schwartz and Boles, 2013; Hill et al., 2014; Bergman et al., 2016; Müller et al., 2017). Thus, it may be that changes occurring at different ages in the microbiome (dysbiosis) contribute to the development of AD.

Very recently, the involvement of fungi in AD has been demonstrated, following long-held suspicion that they may play a role (Pisa et al., 2016, 2017; Alonso et al., 2017). One of the most constantly mentioned mycetes is *Candida albicans*, which has been directly demonstrated in the brain of AD patients. Many other fungi have also been incriminated, the most frequent being the genus *Malassezia*. *Malassezia* are commensals of the oral cavity and also found on the skin. They may become pathogenic to humans in an opportunistic manner. So, most of the fungi originate from the nasal or the oral cavity. These disseminated mycoses may be implicated either as causative agents or as risk factors for AD. Interestingly, fungal products such as polyglucans, β-tubulin, enolase, and chitinase may also be found in the blood and the CSF from AD patients (Pisa et al., 2016). These are part of the body mycobiome (community of fungi inside an organism). However, it is important to mention that fungi are not only found in the brains of AD patients but also in normal and MCI brains. Thus, the question is once more how and why they may become pathogenic and lead to AD. Again, most probably genetic–environmental interactions will make them more or less virulent. Moreover, the number of fungi found, as well as their association with other microbes in senile plaques/biofilms may be important.

Even though there is very strong evidence that the brain of AD patients commonly contains several microbes this is clearly not enough to prove that AD is an infectious disease. It is now well-accepted that in the brain there is a large microbial biodiversity. Most of these organisms have been found in post-mortem brains and documented in many countries throughout the world. So, this is a worldwide phenomenon. Also, this may emphasize the concomitant role of microbial products as triggers of the inflammatory status in the brain. One piece of the puzzle is still missing.

## New Role for Aβ

This missing link came from the seminal work of the group of Tanzi and Moir demonstrating that Aβ is an AMP (Soscia et al., 2010), as shown by comparing Aβ to LL37 (a powerful antimicrobial agent active against various bacteria and fungi). They tested Aβ as AMP against a large number of pathogens (e.g., *Enterococcus faecalis, S. aureus, C. albicans*, etc.). We found that Aβ also has antiviral activity, secreted upon viral infection with HSV-1 (Bourgade et al., 2015, 2016a,b). Indeed, Aβ creates pores in cellular membranes and thus kills bacteria, fungi, and enveloped viruses (Bode et al., 2017).

These observations were followed by the *in vivo* testing of the antimicrobial activity of Aβ. Mice infected with pathogens (e.g., *Salmonella typhimurium*) were intracerebrally treated with Aβ which almost totally prevented infection and plaque formation (Kumar et al., 2016). So, this confirmed the physiological antimicrobial activity of Aβ.

The demonstration that Aβ is an AMP was instrumental in making a link between the infection hypothesis of AD and the Aβ hypothesis of AD. This clearly indicates why trials that targeted Aβ failed and why Aβ, *per se*, could not be the cause of AD (Ferrer et al., 2004). This was followed by reassessment of the nature of senile plaques.

## New Assessment of Senile Plaques

In view of our infection hypothesis it can be said that the plaques in the brain of AD patients and even in the earlier stages may actually be biofilm.

A biofilm is a polysaccharide, protein, nucleic acid conglomerate secreted by one microorganism or by a synergistic microbial community (Costerton et al., 1995; Sapi et al., 2012; Serra et al., 2013; Kriebel et al., 2018). It surrounds the bacteria (or fungus) that secreted it and protects it from desiccation, toxic substances, antimicrobials, or immune attack. Within the biofilm, microorganisms can communicate via quorum sensing (Srinivasan et al., 2017). Many substances may be found inside the biofilm depending on the type of pathogen and the surrounding substances. Thus, microorganisms in biofilms show elevated tolerance to stress and antibiotics as well as to immune mediated attacks conferring to this whole structure an ideal niche to assure the persistence of the microorganism in the environment and mainly in association with the host.

When considering the infection hypothesis and the antimicrobial role of Aβ, it is suggested that senile plaque found in the brain is in fact a biofilm assuring the survival of various pathogens (polymicrobial). This was proposed by the group of Miklossy for treponema (Miklossy, 2016) and by the group of Balin for the *C. pneumoniae* (Balin et al., 2008). The group of Itzhaki also found evidence of HSV-1 DNA in plaques (Wozniak et al., 2009). Indeed, biofilms often contain microbial nucleic acids.

It is important to understand what the composition of senile plaque (biofilm) is. In biofilms, curli fibers, which are microbial amyloids, aggregate and acquire Aβ-like conformations and act as cross-seeding molecules to propagate (Taylor and Matthews, 2015). Indeed, amyloid fibers are abundant in the bacterial world and are recognized as major structural and functional extracellular matrix components of environmental and pathogenic biofilms (Müller et al., 2017). Thus, it would be conceptualized that these microbial products which constitute the skeleton of the biofilm also incorporate Aβ in the brain which will result ultimately in senile plaques (Torrent et al., 2012; Bergman et al., 2016; Müller et al., 2017). As already mentioned, Aβ is an AMP so it is somehow counterintuitive to imagine that Aβ is used by the microorganism for its own destruction unless its incorporation into biofilm leads to its inactivation. In this case, it would not be surprising that microorganisms inactivate host antimicrobial agents. Alternatively, most research has mainly found residues of infectious agents but not live organisms in the AD brain. Thus, senile plaques may be a sort of cemetery for the various microorganisms, containing mainly very toxic and inflammatogenic microbial substances which may stimulate the surrounding microglia. However, it can be imagined that microorganisms may survive in some form that is not sensitive to Aβ attacks (perhaps within biofilms) allowing them to reactivate periodically.

Thus, presently all evidence converges to consider plaques as biofilms. If they may be found, as in some elderly, without dementia, this means that the biofilms may have efficiently contained the microorganisms and no clinical manifestations arise. When the microorganisms exit from the biofilms on periodic reactivation clinical symptoms may appear. These considerations make biofilms extremely important as targets or protectors against therapy. Nevertheless, despite the compelling evidence that senile plaques are biofilms this still does not definitively answer the fundamental question of whether microbes are the causative agents of AD.

## Role of Neuroinflammation and the Innate Immune System

Neuroinflammation is considered a hallmark of AD (McGeer and McGeer, 2013; Bolós et al., 2017; McManus and Heneka, 2017; Pritchard et al., 2017). The infection hypothesis provides the stimulus for this neuroinflammation. It also sheds light on two other fundamental issues: (1) Neuroinflammation may not be entirely the consequence of Aβ deposition (as stated by the amyloid hypothesis) but rather may itself cause Aβ deposition as a protective response toward microbial challenge; and (2) It is not the case that Aβ is exclusively a “harmful” molecule that aggregates to form plaque, but it is also a basic element of the innate immune defense system and thus a “beneficial” molecule as well, at least under certain conditions (Le Page et al., 2018). Ultimately, as reactivations of infections become more frequent and chronic production of Aβ increases, its antimicrobial effect may be blunted by loss of active Aβ through recruitment to plaque formation; inflammation becomes chronic and ultimately deposition proceeds and results in senile plaque formation (Bourgade et al., 2016a,b). The deposition of plaque may be the initiator of the inflammatory process, maintain it and finally destroy the neighboring neurons. This process becomes visible clinically when a threshold is crossed.

Thus, these considerations also suggest that the innate immune system plays an important role in the development and progression of AD not only in the brain but also outside the brain (Le Page et al., 2018). The infection will stimulate the innate immune system as participating cells join forces to eradicate the infections. There will be a successive activation of NK cells, neutrophils, and monocytes/macrophages all undergoing differential activation at different disease stages (from symptom-free to the MCI stage, then to fully developed AD) (Saresella et al., 2014; Le Page et al., 2015, 2017). Aβ is an actor but also a stimulus for the innate immune system, subsequently to other stimuli such as the microbial products found in the brain and in the periphery including LPS, curli products, glucans which act as PAMPs. They induce activation via PRR including TLRs and NODs resulting in a proinflammatory milieu with pro-inflammatory cytokines and chemokines, mainly TNFα, IL-1β, and IL-6 (McManus and Heneka, 2017). Indeed, an increased production of IL-1β and IL-18 by monocytes/macrophages was demonstrated after LPS stimulation reflecting the stimulation of the NLRP3 and caspase-1 pathways (Minogue, 2017; Wohleb and Delpech, 2017). Moreover, they can modify the phenotypes of the microglia as they transform them into M2 phenotypes. Thus, the innate immune system, by inducing neuroinflammation, plays a pivotal role in AD pathogenesis both in the periphery and after migrating to the brain through the increased BBB permeability.

## How May All These Theories Be Reconciled?

The existing data suggest that AD results from a progressive accumulation of noxious inflammatory processes or events in the brain fuelled by multiple infectious agents that colonize/infect the body. Local neuroinflammation may continue at a low level throughout life with little negative effect. However, when exacerbated by reactivation of infections combined with other insults (e.g., oxidative stress), including age-related insults like increasing amounts of senescent cells, the acute inflammatory response results in unbalanced production of cytotoxic mediators, such as TNFα, which, accompanied by immunosenescence/inflamm-aging, becomes difficult to control or stop (Fulop et al., 2018). Microbial metabolites may not only fuel neuroinflammation, but also contribute to senile plaque formation when their biofilm components are integrated into plaque. The enhanced neuroinflammatory process damages neurons and alters the (BBB). These mediators also induce peripheral inflammation and then return to further stimulate local neuroinflammation (Blach-Olszewska et al., 2015; Festoff, 2016; Busse et al., 2017). This progressive pro-inflammatory situation is exacerbated with age, creating a vicious cycle of local and systemic inflammatory responses leading to activation of cytotoxic microglia, unbalanced cytokine production, Aβ accumulation and irreversible brain damage.

## What Is the Required Research Agenda to Test the Infectious Hypothesis?

We suggest that many microorganisms may together cause overproduction of Aβ by affected neurons while their biofilms enhance and reinforce senile plaque and thus they may be the actual cause of AD. By being able to detect them and their biofilms it should be possible to associate them with the development and progression of AD. An important question is how a chronic infection, potentially involving several microorganisms, may smolder for decades, its neuroinflammation remaining silent, and then manifesting itself clinically only decades later. Our preliminary results suggest that an infectious reservoir might exist inside the brain and/or in the periphery, which would be transmitted to the neurons. Reactivation of chronic infection may occur periodically under various stresses, but it is silenced by immunity until immune-surveillance is overcome or a threshold is reached. These chronic infections and reoccurrences would be favored by the presence of biofilms, which assure the survival of the infectious agent in the brain. Biofilm deposition in the brain may also contribute a framework for Aβ to create senile plaques. Indeed amyloid-like proteins made by bacteria form the basis of biofilms. Furthermore, polymicrobial biofilms are generally more robust than those made by individual bacteria and the addition of Aβ might make even stronger senile plaque/biofilm. Thus, detection of infection as early as possible, decades before the clinical manifestations may constitute a proof of infectious etiology.

## Therapeutic Implications of the Infection Hypothesis

It would be very optimistic to imagine that an antibiotic therapy may be administered and cure, or at least prevent progression of AD. It is clear, however that no antibiotic regimen has been developed to cure periodontitis which has a similar implication of chronic infectious microorganisms and biofilms. However, accepting that senile plaques have biofilm components opens avenues to different possibilities to attack the integrity of the biofilm. Small molecules, vaccines and other means could be developed to target the biofilms or the microorganisms that created them. It can be also imagined that by recognizing the infectious etiology of AD, we will be able to discover early markers of the disease and we could develop new prevention trials targeting new molecules. It is clear that Aβ should no longer be considered as the best or only promising target for the prevention and treatment of AD.

## Conclusion

**FIGURE 1:**
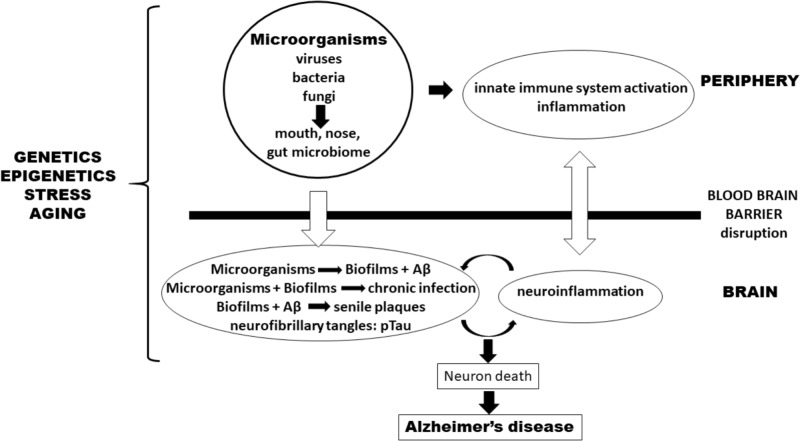
Pathogenesis of Alzheimer’s disease (AD) according to an infection hypothesis incorporating the old amyloid beta hypothesis. Peripheral microbiomes are the source of microorganisms. In the periphery, they activate the innate immune system and cause inflammation which favors penetration of microorganisms either via the blood-stream or transported by monocytes/macrophages through the blood–brain barrier (BBB) into the brain. In the brain they form biofilms which integrate Aβ (pathogen-induced) to form senile plaques. Disruption of the BBB comes both from outside the brain via peripheral inflammatory mediators as well as from within the brain by the cytokines and inflammatory cells having already penetrated the BBB as well as mediators generated by microglia that have been stimulated by chronic infections. Genetics/epigenetics, stress, and aging affect both sides. Together they form a vicious circle, originating (and perpetuated) by chronic infections or pathogen reactivation. An additional smaller amplifying circle exists between plaques and biofilms which stimulate neuroinflammation in the brain. This process leads to neuron death and eventually to AD.

Many experimental data support the involvement of a polymicrobial community in the pathogenesis of AD. This “neurobiome” would be the backbone to support the new infection hypothesis of AD (**Figure 1**). This new hypothesis naturally incorporates the old amyloid hypothesis as Aβ is again on the center stage but as a puppet to infectious masters, rather than as a principal actor. This also throws a new light on neuroinflammation which precedes Aβ production and is not a consequence of it. So, in our view the question is not whether the microorganisms are the causative agents of AD but how and when they are inducing it. How all these mostly commensal microorganisms became pathological and when in the evolution of the disease they will manifest themselves remains to be discovered. It can be assumed that advancing age may be a common driving factor to explain the how and the when by influencing either virulence factors or accumulation as well the progressively more senescent defense state against the infection which had installed itself perhaps decades earlier. This pathological situation is not without parallel to the natural history of cardiovascular diseases which also develop via atherosclerotic lesions that appear decades before clinical manifestations. The age-associated loss of control of (neuro) inflammation will also play a role in AD. This new hypothesis generates hope to find new targets, even if biofilms are difficult to combat as reported in the case of periodontitis. Nevertheless, a better understanding of the role of the “neurobiome” will ultimately result in the prevention and treatment of this disastrous disease.

## Author Contributions

TF, JW, KB, AK, EZ, AL, KH, GP, CB, GL, GD, and EF discussed and contributed to write the article. TF and EF conceptualized the article.

## Conflict of Interest Statement

TF has received consultation fee from Eisai, Co. The remaining authors declare that the research was conducted in the absence of any commercial or financial relationships that could be construed as a potential conflict of interest.
